# Preparation of UiO-66 loaded Letrozole nano-drug delivery system: enhanced anticancer and apoptosis activity

**DOI:** 10.1186/s13568-024-01689-1

**Published:** 2024-04-15

**Authors:** Maryam Ronaghi, Ramtin Hajibeygi, Reza Ghodsi, Akram Eidi, Ronak Bakhtiari

**Affiliations:** 1 Department of Biology, Science and Research Branch, Islamic Azad University, Tehran, Iran; 2 Advanced Diagnostic and Interventional Radiology Research Center (ADIR), Tehran University of Medical Science, Tehran, Iran; 3 Department of Chemical and Petrochemical Engineering, Sharif University of Technology, Tehran, Iran; 4 Department of Pathobiology, Division of Microbiology, School of Public Health, Tehran University of Medical Sciences, Tehran, Iran

**Keywords:** UiO-66, Letrozole, Drug release, Cytotoxicity, Apoptosis

## Abstract

The use of drug delivery systems in targeting and achieving the targeting of drugs in treating diseases, especially cancer, has attracted the attention of researchers. Letrozole is one of the drugs for the treatment of breast cancer. In this study, the organic-metallic pharmaceutical porous nanostructure based on zirconium UiO-66 loaded letrozole was synthesized. Its cytotoxicity and effect on apoptosis and migration against breast cancer cell line were investigated. In this experimental study, the UiO-66 nanoparticle-loaded letrozole was synthesized using zirconium chloride (ZrCl4), dimethylformamide (DMF), and HCl. Its characteristics were determined by scanning electron microscopy, and its average size was determined by the DLS method. Also, the rate of letrozole drug release from the nanoparticle was investigated in 24, 48, and 72 h. In addition, its cytotoxicity effects were investigated using the MTT colorimetric method at concentrations of 3.125-100 µg/ml against the breast cancer cell line (MCF-7) in the periods of 48 and 72 h. Also, the expression level of apoptotic genes *Bax* and *Bcl2* was investigated by the Real-Time PCR method. Also, the amount of cell migration was done by the migration assay method. The results showed that UiO-66 bound to letrozole had a spherical morphology and an average size of 9.2 ± 160.1. Also, the letrozole drug was loaded by 62.21 ± 1.80% in UiO-66 nanoparticles and had a slower release pattern than free letrozole in the drug release test, so within 72 h, 99.99% of free letrozole was released in If in UiO-66 containing letrozole, 57.55% of the drug has been released. Also, the cytotoxicity results showed that UiO-66 bound to letrozole has more significant cytotoxic effects than free letrozole (*p* < 0.05). Also, the results of Bax and Bcl2 gene expression showed that the treatment of MCF-7 cells with UiO-66 nanoparticles attached to letrozole increased the expression of Bax and Bcl2 genes compared to the reference gene Beta-actin in MCF-7 cell line, respectively. (*p* < 0.05) increased by 3.71 ± 0.42 and (*p* < 0.01) decreased by 0.636 ± 0.034 (*p* < 0.05). Cell migration results showed that the concentration of 50 µg/ml of UiO-66 bound to letrozole decreased the migration of MCF-7 cells. Generally, the results of this study showed that UiO-66 loaded letrozole can be used as a suitable drug carrier for cellular purposes, as it has increased the effects of cytotoxicity and the rate of apoptosis in breast cancer cell line (MCF-7), so it can be used with more studies used nanocarriers as a drug delivery system.

**Supplementary Information:**

The online version contains supplementary material available at 10.1186/s13568-024-01689-1.

## Introduction

Breast cancer is one of the major causes of death in the world due to the lack of an effective drug that would cause fewer side effects (Waks and Winer 2019). However, there have been significant improvements in the field of cancer by the exertion of new techniques in designing drug delivery systems (Tiwari et al. 2012). Novel pharmacological systems have proved to be effective in the targeted and specialized treatment of cancer and have provided a new field that involves the usage of porous materials for delivery systems (Dinda and Pattnaik 2013; Amale et al. 2021; Hojabri et al. 2023). Letrozole is a type of chemotherapy drug that is currently approved for the treatment of colorectal cancers. However, the in vivo anti-tumor activity of letrozole has been reported to be low when used alone (Lisztwan et al. 2008). This low activity can be attributed to the high partitioning of red blood cells and low drug accumulation in tumor tissues after intravenous injection. On the other hand, the clinical efficacy of this drug is limited by its side effects, such as neurotoxicity, which are also dose limiters (Miller et al. 2002; Pourmoghadasiyan et al. 2022). Side effects of letrozole and other chemotherapeutic drugs are the result of systemic administration and, as a result, the effect of these drugs on normal cells in addition to cancer cells (Simpson et al. 2004). Considering the above, it seems necessary to use a new drug delivery system that can reduce the side effects of the drug by entering the tumor site and increasing its anti-tumor activity by preventing it from entering normal tissues (Rabiei et al. 2020; Gholami et al. 2022). Therefore, one of the ways to overcome these challenges is the targeted delivery of chemotherapy drugs using nanoparticles. (Lázaro and Forgan 2019). In this work, the fabrication of UiO-66-NH_2_ nanomaterials containing letrozole is considered for inactive targeting. With the remarkable nanoscience development that has been created over the past few decades, porous nanomaterials have been developed that can conjointly alleviate various of the aforementioned side-effects when combined via letrozole into novel drug delivery systems (DDSs) (Horcajada et al. 2010). Between the porous substances, metal-organic frameworks (MOFs) are the most flexible in terms of their design flexibility and applicability as novel nanomaterials-based DDSs (Nilash et al. 2019). The various toxicological shapes that recently introduced nano MOFs displayed negligible toxicity profiles for carboxylate-based nano MOFs.

Novel nanoparticle-based hybrid materials, such as metal-organic frameworks (MOFs), have been investigated for drug delivery applications. MOFs are low-density crystalline porous materials with a large internal surface area composed of metal ions or clusters (nodes) and organic bridging ligands in 2D or 3D configurations (Butova et al. 2016; Molani et al. 2019). MOFs offer promising features like a large surface area, unique morphology, high pore volume, customizable components, functionalized pore surfaces, superior porosity, and diverse topologies for various applications. In comparison to traditional drug carriers, MOFs offer advantages like numerous pores and specific surface areas for loading therapeutic or diagnostic agents, as well as the ability to modulate physical and chemical properties through metallic clusters and organic ligands. However, challenges such as low biocompatibility, inadequate chemical and thermal stability, and premature drug release have limited the drug adsorption and delivery capabilities of MOFs. Despite these challenges, MOFs have been extensively studied as potential drug carriers due to their distinct properties. Different MOFs exhibit varying drug loading capacities and release profiles based on their specific ligands (Sun et al. 2013). Zirconium-based MOFs, particularly the well-known Zr terephthalate UIO-66 structure, have garnered significant attention for drug delivery applications. UIO-66 is a MOF structure comprising [Zr6O4(OH)4] octahedron clusters and Ben-zene-1,4-dicarboxylic acid (BDC) ligands. Various derivatives of UIO-66 with different functional groups have been utilized for encapsulating anticancer agents owing to their exceptional chemical and physical properties like excellent water resistance, superior chemical, thermal, and mechanical stability, ideal biodegradability, and high specific surface area (Cai et al. 2020; Salekshahrezaee et al. 2021; Nasrabadi et al. 2019).

In this study, letrozole was integrated into the structure of UiO-66-NH2 and applied in vitro to deliver letrozole to the breast cancer cells (Meng et al. 2021). The performance of this structure was investigated using MTT assay and Real-Time PCR.

## Materials and methods

### Materials

Trypsin-EDTA, Trypan blue, Medium RPMI-1640, DMEM, PBS, FBS, MTT, and Penicillin/Streptomycin 100 X were purchased from Gibco, USA. Dialysis membrane (MWCO 12,000 Da). Congo red, crystal violet solution, and all the other chemicals were purchased from Sigma-Aldrich Chemicals (St. Louis, MO). All the media were purchased from HiMedia Laboratories, India; the control bacterial strain was purchased from the American Type Culture Collection (ATCC). HFF cell lines were obtained from Pasteur Cell Bank, Iran. RNA extraction kit was purchased from Qiagen, United States. Revert First Strand cDNA Synthesis Kit (Fermentas, Lithuania) was applied to synthesize the cDNA. All other chemicals and analytical grade solvents were also supplied from Merck (Germany).

### Preparation of UiO-loaded letrozole

**Scheme 1:**
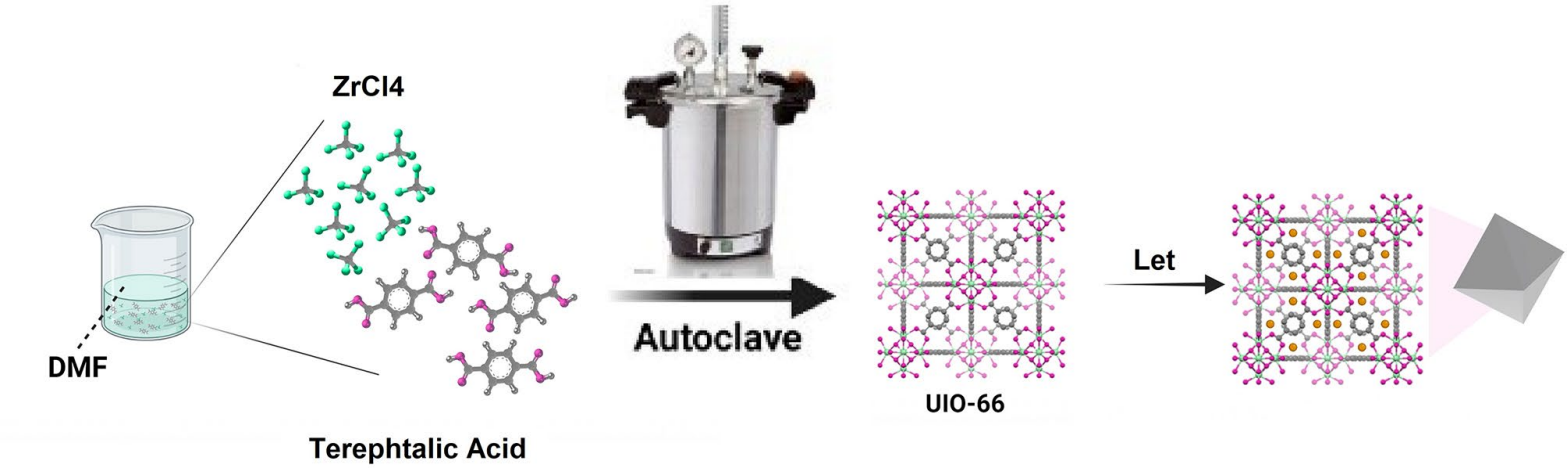
Preparation of UiO-66, and UiO-66-Let

In this research, 0.52 g of 2-amino terephthalic acid was removed and dissolved in 40 ml of N, N Dimethylformamide (DMF). Then 0.5 g of zirconium chloride salt (ZrC14) was dissolved in 20 ml of dimethylformamide (DMF) and 4 ml of HCl in a sonicator bath separately. The two resulting solutions were mixed and placed in the sonicator bath for 20 min to complete the sonication process. The obtained solution was heated at 80 °C for 36 h. Then, it was placed in a centrifuge at 3000 rpm to carry out the sedimentation process. The sediment obtained was kept at room temperature and then placed at 36 °C for 6 h to dry. To load letrozole into UiO-66, 5 mg of UiO-66 was mixed with 10 mL of letrozole drug (1 mg/mL) and placed in a shaker for 24 h. Finally, the UiO-66 solution attached to letrozole was washed twice with distilled water and then placed in a centrifuge at 12,000 rpm for 20 min (Scheme 1)(Zhang et al. 2022).

#### Encapsulation efficiency

Encapsulation efficiency (EE%) refers to the drug encapsulated in the UiO structure compared to the primary drug used. For this purpose, the UiO formulation was centrifuged at 4 °C and 14,000 g speed for 45 min. UiO-loaded letrozole is precipitated, and free UiO remains in the supernatant. The absorbance of the supernatant sample was read by a spectrophotometer at a wavelength of 265 nm, and the amount of free letrozole was calculated and subtracted from the initial amount of the initial letrozole, and from that, the EE% was calculated:

1 $$\begin{aligned} & \text{Encapsulation efficiency}=\text{amount of free drug} \\ & \,- \text{the amount of primary drug}\\ &\,/\text{amount of primary drug}\times 100 \end{aligned}$$

### Drug release test

Drug release is evaluated dynamically. In this way, 2 ml of UiO-loaded letrozole and free letrozole are placed in the dialysis bag (molecular weight cutoff 12 KDa) separately. Each of the bags is suspended in a flask containing 50 ml of phosphate-buffered saline (PBS) at 37 °C. The flasks with the bag containing the UiO-loaded letrozole and the free letrozole are placed on the stirrer. Sampling is done at different hours in such a way that 1 ml of PBS containing the dialysis bag is removed, and 1 ml of PBS with a temperature of 37 °C is replaced. Sampling is done up to 72 h in specific time intervals (1, 2, 4, 8, 24, 48, and 72 h). The optical absorption of the samples was read by the UV spectrophotometer at a wavelength of 265 nm and the graph of the cumulative release percentage of letrozole from UiO was drawn for 72 h (Sadek et al. 2022).

### Cell toxicity and biocompatibility test

Cytotoxicity of UiO-loaded letrozole and free letrozole was studied using a colorimetric assay (MTT) against three breast cancer cell lines (MCF-7). For this purpose, cell lines were obtained from the Pasteur Institute Iran cell bank of Iran (Tehran-Iran). Then, the cells were seeded separately at the density of 10^4^ cells per well in a 96-well plate for 24 h. Then, the cells were treated with different concentrations of UiO-loaded letrozole and free letrozole (3.125 to 100 µg/ml), and after 48 h of incubation, MTT dye solution (5 mg/ml in PBS) was added to the wells and kept in the incubator for 3 h. The supernatant solution was removed, and 100 µl of dimethyl sulfoxide (DMSO) solution was added to it, and the absorbance of all the wells was read at 570 nm wavelength, and according to the following formula, the survival percentage of the cells was calculated (Jarai et al. 2020):

2 $$\begin{aligned} & \text{Cell survival} = \text{optical absorbance of treated cells}\\& \,/ \text{optical absorbance of control} \times 100 \end{aligned}$$

#### Expression analysis of apoptotic genes

In this study, to investigate the expression of apoptotic genes including *Bax* and *Bcl2*, it was measured by the Real-Time PCR method. At first, the total RNA of treated and untreated cells with UiO-loaded letrozole and free letrozole was extracted using the RNA extraction kit (Qiagen, USA) according to its instructions. The synthesis of complementary DNA was done with a cDNA synthesis kit (Fermentas, Lithuania). To perform Real-Time PCR, the specific primers of target genes *Bax*, *Bcl2*, and beta-actin gene (Housekeeping gene) were used as an.

internal control. The reverse primer sequence of the *Bax* target gene was 5’-TTGCTTCAGGGTTTCATCCAG − 3’, and reverse 5’- AGCTTCTTGGTGGACGCATC − 3’. The reverse primer sequence of the *Bcl2* target gene was 5’-TGTGGATGACTGAGTACCTGAACC-3’, and reverse 5’-CAGCCAGGAGAAATCAAACAGAG, and for beta-actin reference gene as 5’-CGTCTGCCCTATCAACTTTCG-3’, and reverse 5’-CGTTTCTCAGGCTCCCCTCT-3’ (Chen et al. 2021).

### Migration assay

For the migration assay, MCF-7 cells (5 × 104 per well) were seeded in the upper chamber with serum-free medium, while the lower chamber was filled with 500 µL of medium with 20% FBS. Following incubation with the samples of interest for 72 h (letrozole and UiO-66 loaded letrozole), cells on the lower surface of the membrane were stained with crystal violet. Cells were counted under a microscope to investigate cell migration (Wu et al. 2022).

### Statistical analysis

All the tests of this study were repeated 3 times and the results were analyzed by GraphPad Prism (version 8) software using a one-way analysis of variance, and *p* < 0.05 was considered significant.

## Results

### Characterization of UiO-loaded letrozole

**Fig. 1:**
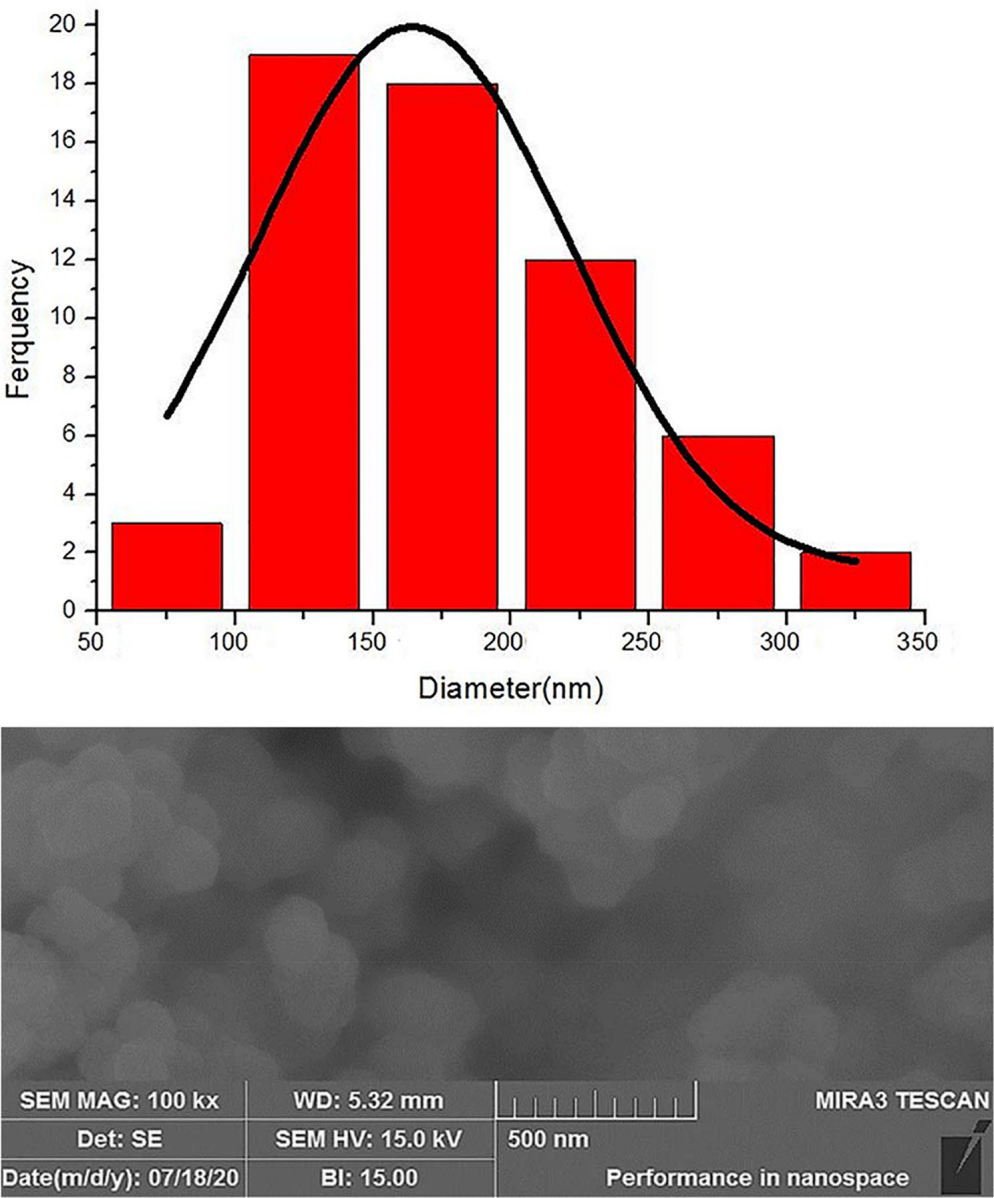
The synthesized UiO-loaded letrozole characteristics. (**A**) SEM of UiO-loaded letrozole, (**B**) Average size chart

The SEM and DLS were used to investigate the morphological characteristics of the synthesized UiO-loaded letrozole. The results of the SEM show that the synthesized UiO-loaded letrozole has a spherical structure (Fig. 1A). Also, the DLS results showed that the optimal size of the synthesized UiO-loaded letrozole is 160 nm (Fig. 1B). Also, the nanoparticle size chart shows that the size of the synthesized nanoparticles was between 50 and 350 nm, and the average size of the nanoparticles was 160 nm (Table 1).

**Fig. 2:**
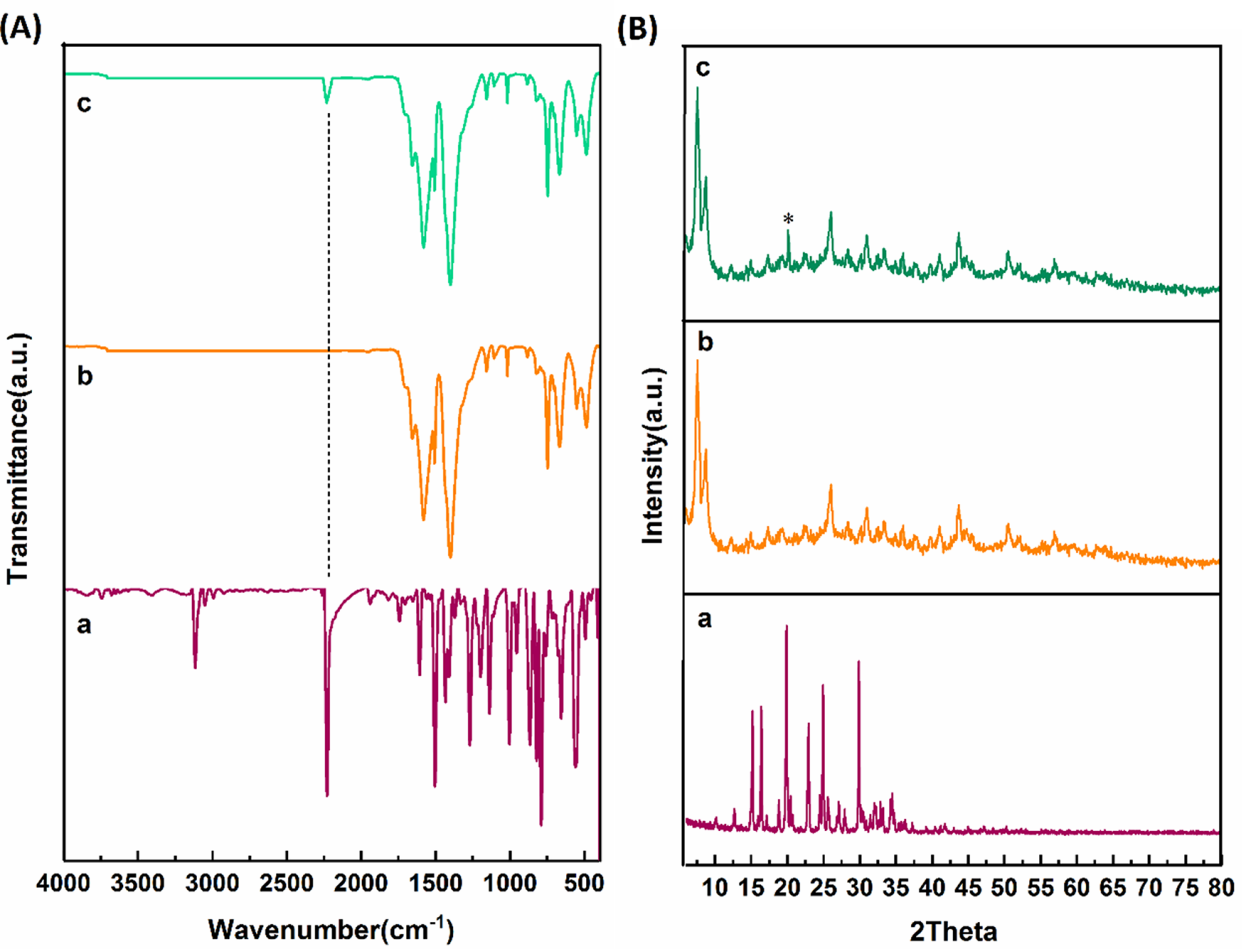
(**A**) FTIR results of a) Let, (b) UIO-66, and (c) UIO-66-Let; (**B**) XRD results of (a) Let, (b) UIO-66, and (c) UIO-66-Let.

All bands related to UIO-66 and UIO-66-Letrozole linkages are given in Table S1. When the letrozole drug was incorporated into the UIO-66 structure, a medium and sharp absorption band related to the C ≡ N bond appeared in the 2232 region, which can confirm the successful loading of letrozole into the UIO66 structure (Fig. 2A). The peaks related to UIO-66 appeared at angles of 7.56, 8.68, 12.15, 14.90, 17.30, 19.17, 22.63, 25.95, 27.58, 30.17, 31.08, 33.46, 35.87, 37.71, 39.72, 40.91, 43.77, 44.77, 50.57, 51.94, 57.01. The peaks at 7.56, 8.68, and 25.95 angles can be called characteristic peaks. When letrozole was added, all the peaks related to the UIO-66 structure were accompanied by a slight shift towards smaller angles, which may indicate an increase in the interlayer space of the structure and the entry of letrozole into the structure. In addition, the peak related to letrozole appeared at an angle of 20.11 degrees (marked with an asterisk), which can confirm the presence of letrozole in the structure (Fig. 2B).

**Table 1:** Physical characteristics of synthesized nanoparticles

Parameter	UIO-66	UIO-66-Let
Average size (nm)	116.1 ± 6.5	160.1 ± 9.2
PDI	0.132 ± 0.013	0.188 ± 0.017
Entrapment Efficiency (EE) (%)	--	62.21 ± 1.88

### Drug release pattern

**Fig. 3:**
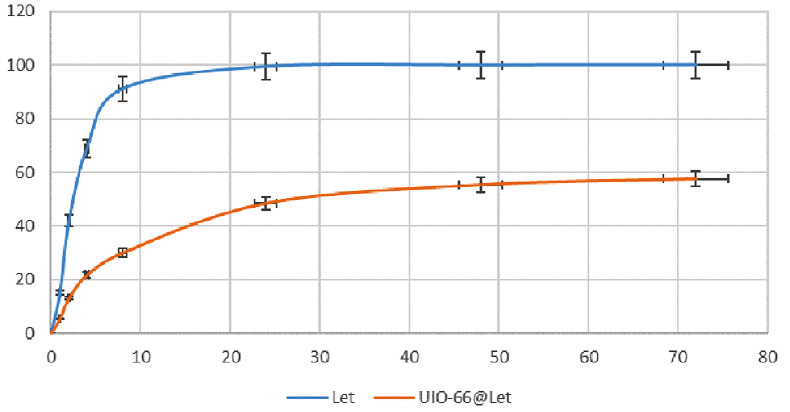
The release profile of letrozole from UiO-loaded letrozole at 72 h. letrozole release from UiO form has been compared with free letrozole

Figure 3 shows the cumulative release process of the free form of the letrozole and the UiO-loaded letrozole in the release medium of PBS for 72 h. To simulate and bring the ex-vivo release environment closer to the real and in vivo conditions, the PBS release medium was used for the receptor phase, the release of the letrozole from the UiO form (57.55%) is less than the free letrozole (100%) during the release period (72 h). In the release of free letrozole, 93% of the letrozole was released in the medium during the first 8 h, but for the UiO-loaded letrozole, 30% of the letrozole was released from the UiO form within 8 h of release. According to the results, drug release from UiO occurs in 2 steps. The initial stage of 0–8 h, when the release of letrozole is very fast and explosive, diffuses letrozole to the release medium. The second stage is the slow-release stage, in which letrozole slowly diffuses to the release medium for 72 h. In general, studies show that drug release from UiO is explosive in the first hours (first 8 h) and gradually decreases in the following hours.

### In vitro cytotoxicity

**Fig. 4:**
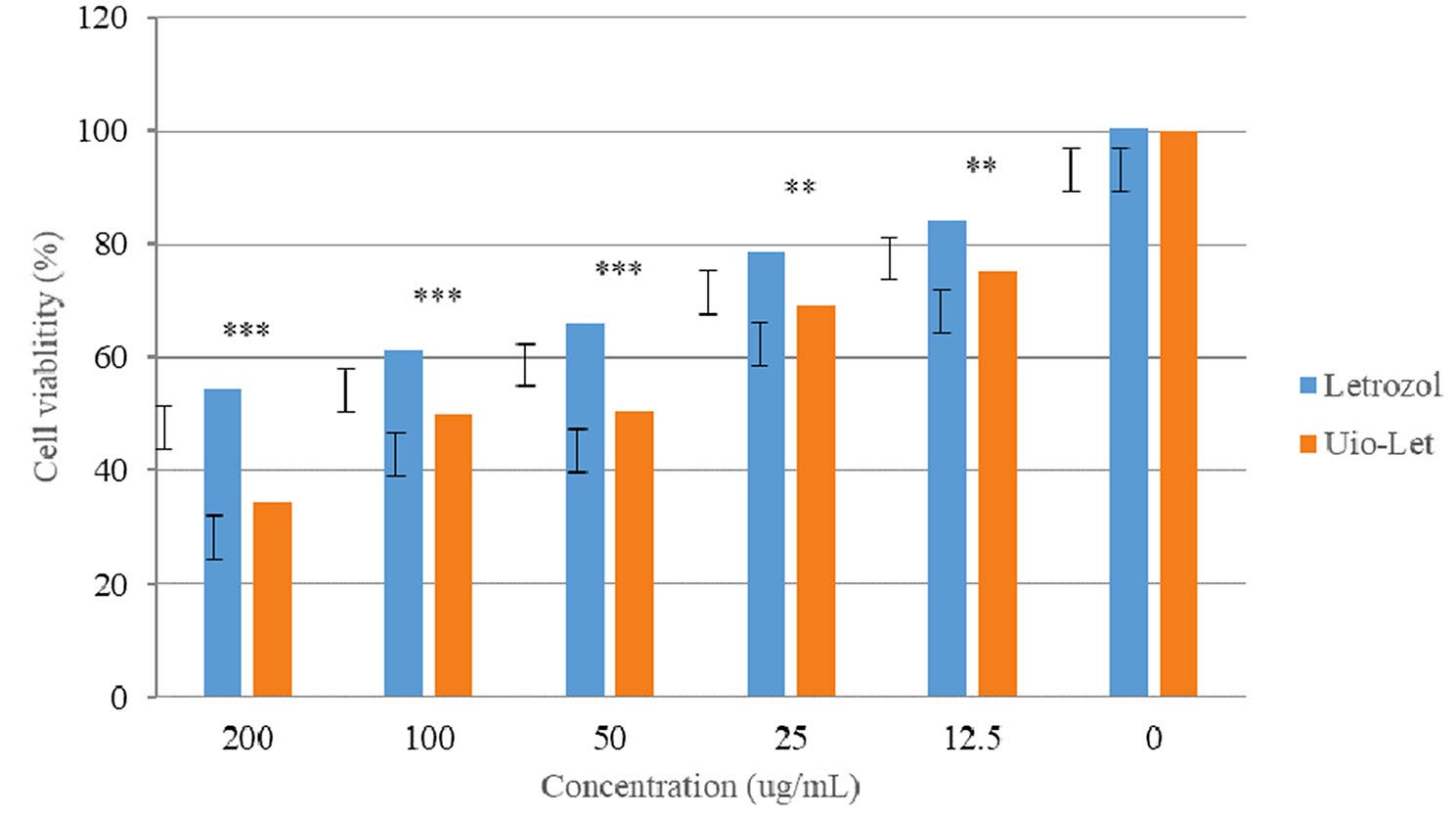
In vitro cytotoxicity of free letrozole and UiO-loaded letrozole on MCF-7 cell line. *n* = 3, *** *p* < 0.0001, ** *p* < 0.01

**Fig. 5:**
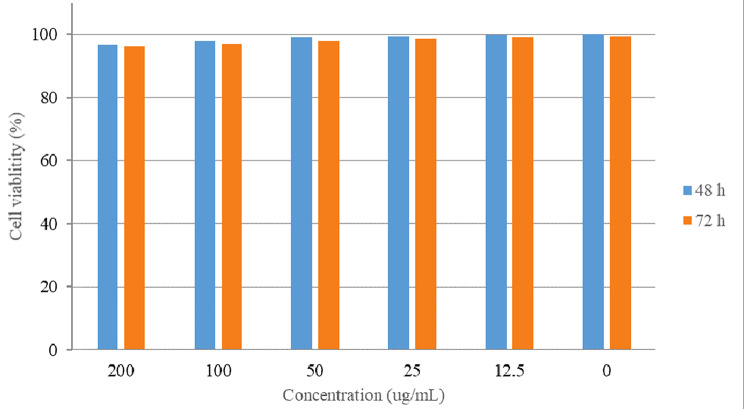
The effectiveness of different concentrations of UiO-66 without drugs in the period of 48 and 72 h. In this graph, the data is expressed as the average percentage of cell viability and standard deviation

The cytotoxicity results of free letrozole and UiO-loaded letrozole on breast cancer cell lines in 24 h showed that UiO-loaded letrozole had the greatest cytotoxic effect in comparison to free letrozole (Fig. 4). The results showed that at the highest concentration )200 µg/ml (, the cell survival rate in MCF-7 cells treated with UiO-loaded letrozole was 54.22 ± 1.21, respectively, which was much lower than free letrozole, which shows the significant cytotoxic effects of UiO-loaded letrozole. Also, the UiO-loaded letrozole had dose-dependent cytotoxicity, and the cell survival rate decreased with the increase in the dose (Fig. 4). MCF-7 cells were treated with different concentrations (200, 100, 50, 25, and 12.5 µg/ml) of UiO-66 without letrozole by MTT method during 48 and 72 h of incubation. According to Fig. 5, the cell viability percentage of the cells in all the concentrations of free UiO used did not have significant cytotoxic effects on MCF-7 cells and indicated the ineffectiveness and safety of the UiO-66 nanoparticle (Fig. 5). Also, using this test, IC50 concentrations were calculated for UiO-loaded letrozole and letrozole, which were 100 µg/ml and 170 µg/ml, respectively.

### Apoptosis gene expression analysis

**Fig. 6:**
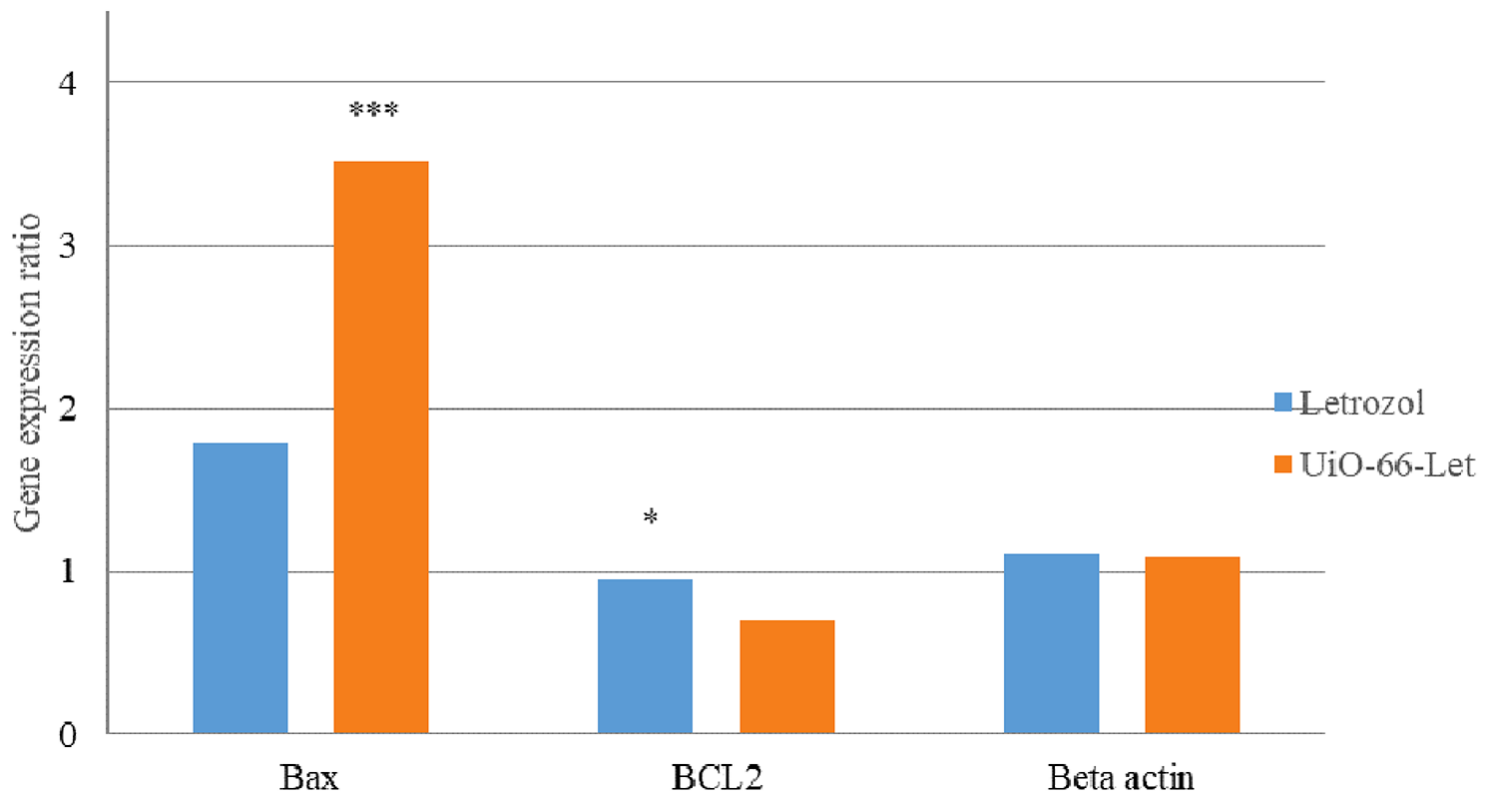
The expression of Bax and Bcl2 genes in cells treated with letrozole and UiO-66 loaded letrozole (* *P* < 0.05, *** *P* < 0.001: *n* = 3)

The expression of apoptotic genes, including Bax and Bcl2, in MCF-7 cells treated with the IC50 concentration of UiO-loaded letrozole and free letrozole, was evaluated using the Real-Time PCR method. The Real-Time PCR data analysis was performed based on the cycle threshold (Ct) comparison. In this study, the difference in the Ct obtained from the tested samples (cells treated) and the control samples (not treated cells) was calculated, and the gene expression was calculated using the ΔΔCt formula (the ratio of the target gene to the reference gene (beta-actin) was calculated through 2 ^−ΔΔCt^). The results showed that the expression of the *Bax* gene compared to the reference gene in the breast cancer cell line was up-regulated significantly within 24 h compared to free letrozole. Also, the level of *Bcl2* gene expression in breast cell lines treated with UiO-loaded letrozole was down-regulated significantly compared to free letrozole (Fig. 6).

### Migration assay

**Fig. 7:**
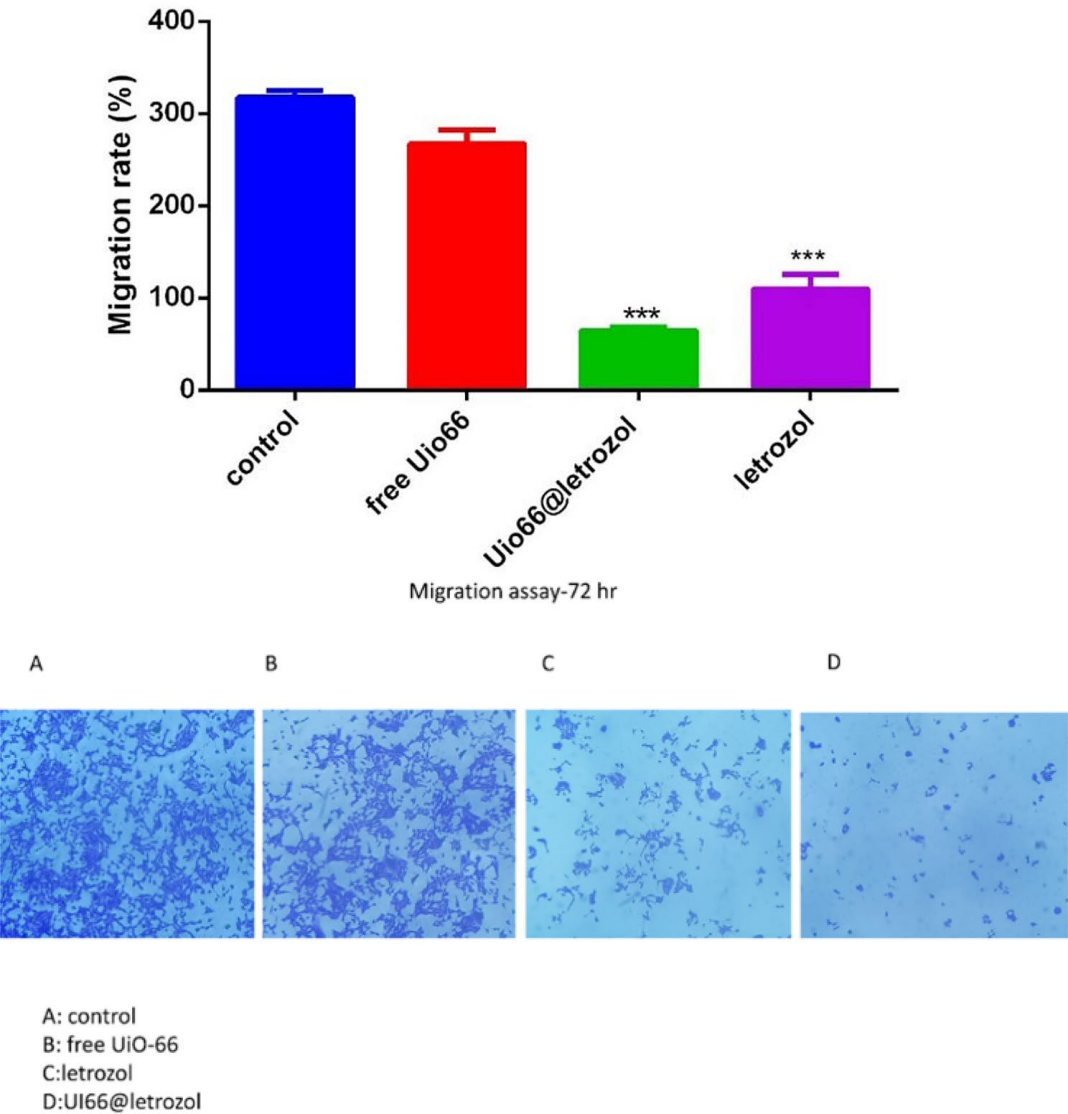
Cell migration test results. (**A**) control, (**B**) MCF-7 cell treated with free UiO-66, (**C**) MCF-7 cell treated with letrozole. (**D**) MCF-7 cells treated with UiO-66 bound to letrozole. The results showed that MCF-7 cells treated with UiO-66 bound to letrozole significantly inhibited cell migration

In this test, the cells of the control group (no treatment) showed faster migration and invasion by filling the scratch area compared to the cancer cells treated with letrozole and UiO-66 bound to letrozole in 72 h. MCF-7 cancer cells treated with a concentration of 50 µg/ml of letrozole by filling the scratch area for 48 h showed little effect on cancer cell migration, While the obtained images showed that the concentration of 50 µg/ml of UiO-66 attached to letrozole decreased the mobility of MCF-7 cells (Fig. 7).

## Discussion

Recently, nanomedicine has made significant progress in cancer treatment; it has minimized the main problems of common treatments (Noorbazargan et al. 2021; Mirzaie et al. 2022; Trushina et al. 2022). The anti-cancer properties of drug-carrying nanoparticles have been proven several years ago, and the use of combining various types of nanoparticles with different anti-cancer drugs has been able to increase the therapeutic effects through synergy and has been proposed as an advanced therapeutic strategy (Mansouri et al. 2021; Haddadian et al. 2022; Asadipour et al. 2023; Hidayat et al. 2023).

Given the rising occurrence of breast cancer within communities globally, chemotherapy stands out as a primary form of intervention; however, it carries significant disadvantages. These include harming healthy cells when administered at elevated and prolonged doses. Additionally, poor water solubility results in limited availability in bloodstreams (bioavailability), liver removal, and lower cellular uptake (Bazzazan et al. 2023). A further constraint is that metastasized cancer cells often exhibit resistance to chemotherapeutics, which contributes significantly to cancer-associated mortality rates. This resistance stems from various cancer cell mechanisms, such as increased drug expulsion, diminished drug intake, enhanced detoxification processes, and augmented DNA repair capabilities. Nanotechnology-driven drug delivery systems offer potential solutions by reducing adverse side effects related to drugs while simultaneously enhancing their therapeutic impact through more efficient drug distribution and heightened treatment effectiveness (Moammeri et al. 2023). To bridge the divide between preclinical toxicity assessments and ensuring patient well-being, nanomedicines present safety concerns across three distinct levels, beyond the inherent toxicity of the drug they contain. Utilizing nanoparticles for delivery often leads to significant changes in how drug molecules are distributed in the body, potentially causing localized overexposure in specific organs upon absorption. A crucial challenge lies in effectively managing the chemistry, manufacturing, and quality control (CMC) aspects of nanomedicines. Unlike traditional medications, the effectiveness of nanomedicines in vivo is paramount, with safety and dependability hinging directly on the physicochemical characteristics of the nanoparticle-encapsulated drugs (Ahmadi et al. 2022; Akbarzadeh et al. 2022). In this study, a UiO-66 nanocarrier attached to letrozole was used to investigate the effects of cytotoxicity against breast cancer cell lines. The idea is that the drug is absorbed into a MOF and then released inside the body at the target site. UiO-66 is a form of zr-MOFs that is resistant to water and heat. UiO-66 is composed of 12 clusters and a linear dicarboxylate linker (Wang and Youle 2009).

When comparing SEM and DLS data of the synthesized NMOFs, the average particle sizes were smaller by SEM. This discrepancy may result from the drying process required for SEM imaging. Specifically, SEM provides an exact measure of each dried nanoparticle’s diameter for determining mean size. In contrast, DLS measures the hydrodynamic diameter, accounting for the nanoparticle core along with any surface-bonded molecules like ions and water. Other studies have made similar observations (Rakhshani et al. 2021).

In this study, the maximum encapsulation efficiency (EE) of letrozole in the synthesized MOF was found to be 62.21%. Due to their high surface area and large pore diameters, MOFs can encapsulate substantial amounts of a targeted drug (Giliopoulos et al. 2020). Providing suitable nanocomposite carriers for slow release over extended periods (Khatibi et al. 2022). Another study encapsulated Cur into UIO-66-NH2, achieving a 7.33% loading capacity and 56.18% EE (Moammeri et al. 2021).

The results of the release align with those of Mocniak et al. Due to this, cisplatin is released much slower from UiO66-NH2 than UiO66, with only 12.5% release after 24 h (Mocniak et al. 2015). Compared to free drugs, the sustained release afforded by MOFs boosts anticancer efficacy and reduces systemic toxicity.

In this study, the cytotoxicity effects of UiO-66 bound to letrozole and letrozole alone at concentrations of 12.5 to 200 µg/ml were investigated using the MTT colorimetric method for 48 and 72 h. One of the reasons that were investigated during the 48 and 72 h of cytotoxicity tests was that the release rate of the drug was higher in these two time periods, and that is why these times were chosen. As shown in the drug release section, in the release of letrozole, 93.89% of the drug was released in the first 8 h, but for the nanocarrier containing letrozole, 30.11% of letrozole was released from UiO-66 within 8 h of release. Finally, within 72 h, 99.99% of letrozole was released, while in UiO-66 containing letrozole, 57.55% of the drug was released. The results of the cytotoxicity test showed that UiO-66 bound to letrozole has lethal effects dependent on concentration and time in breast cancer cells. As the results showed, UiO-66 attached to letrozole had time- and dose-dependent cytotoxicity, so with the increase in time and concentration, the cytotoxicity of UiO-66 attached to letrozole increased, which is mentioned in many studies.

The enhanced cytotoxicity of drug-loaded carriers can be attributed to two key factors: first, the intact MOF structure enables sustained, controlled release to target sites over prolonged periods, and second, intracellular drug concentrations differ since encapsulated drugs enter cells differently than free drugs (Abazari et al. 2018). Danafar et al. reported no cytotoxicity of UIO-66-Cur toward HFF-2 cells, even at high MOF concentrations (Molavi et al. 2018). The results of our study are reasonably consistent with the findings from Farboudi et al., in which they generated UIO-66 NMOFs loaded into carboxymethyl chitosan/polyethylene oxide/polyurethane core-shell nanofibers for sustained co-delivery of DOX and folic acid to cancer cells (Farboudi et al. 2020).

Akbarzadeh et al. prepared the anticancer effect of pegylated niosome formulation containing letrozole-curcumin which was targeted using folic acid and optimized based on the characteristics of size, dispersion index of particles, encapsulation, and release efficiency. The anticancer effect of the optimized and targeted niosome formulation was investigated on MCF-7 and MDA-MB-231 lines, and the results showed that the optimized and targeted form significantly induced apoptosis in the examined cancer cells compared to their free form. In 2019, Wanda Kupta et al. investigated the delivery of docetaxel using UIO-66 nanoparticles targeted with PEG to investigate its toxicity on the MCF-7 cell line. After investigating the amount of drug absorption in the nanoparticle structure and examining its release rate, the cytotoxicity of the prepared formulations was investigated using the MTT method, and they observed that the survival percentage of the cells treated with nanoparticles containing the drug was higher than that of the free drug at the same concentration. Also, targeting the drug-containing nanoparticle did not have a significant effect on the survival of cancer cells compared to the drug.

In general, the induction of programmed cell death is one of the attractive approaches in the nano field. The cell death pathway can involve the activation of mitochondrial organelle proapoptotic events in the cell, which starts with the release of cytochrome c (Kasravi et al. 2023). In addition, the effects of letrozole in increasing the expression of apoptosis genes in cancer cells and inducing programmed cell death have been shown in different studies. Therefore, due to the high level of mitochondrial activity in the respiration process of cancer cells compared to normal cells, a suitable platform is provided for the drug letrozole to destroy cancer cells. Another reason is the morphological differences between the membrane of cancer and normal cells in terms of the difference in the size of their pores (Frandsen et al. 2016). Another aim of this study was to investigate the expression level of apoptotic genes Bax and Bcl2 in cells treated with UiO-66 attached to letrozole and compare it with letrozole alone. The results of this study showed that after the treatment of MCF-7 cells with UiO-66 bound to letrozole, the expression of apoptotic genes significantly increased compared to the reference gene, which indicates the induction of apoptosis in MCF-7 cells. It is important to identify the importance of the expression pattern of this gene in response to the metastasis activity of anticancer drugs. Therefore, more investigations are necessary to prove whether the mRNA expression profile of this gene can be evidence for its role in more accurate and specific prediction of cancer response to treatment.

Bcl-2 can prevent cytochrome c release from mitochondria, significantly inhibiting cell growth (Liang et al. 2009). In this study, letrozole treatment markedly reduced Bcl-2 expression, increasing cell death and exhibiting letrozole-mediated tumor growth inhibition via enhanced apoptosis. Breast cancer cells may more readily undergo apoptosis with upregulation of these genes. The Bcl-2 gene prevents apoptosis by sequestering cytochrome C in mitochondria. Lower Bcl-2 expression can also impact cytochrome C release, allowing it to flow from mitochondria into the cytoplasm. This triggers caspase activation and apoptosis (Jürgensmeier et al. 1998; Gotow et al. 2000). Bax can form oligomers that permeabilize the mitochondrial outer membrane, inducing apoptosis (Westphal et al. 2011). In short, cancer cell growth relies on relative levels of pro- and anti-apoptotic proteins (Yoon and Roh 2012; Wood et al. 2013). When cancer cells are treated with nanoformulations, Bax activation and Bcl-2 downregulation occur. Past research shows high Bcl-2 levels can impart chemotherapy resistance in cancer cells. Therefore, inhibiting Bcl-2 production while increasing Bax production may impede programmed cell death (Garmarudi, Ghasemi et al. 2015, Sartorius and Krammer 2002, Singh et al. 2019). The study found that UIO-66-letrozole was more effective in inhibiting cell migration than free letrozole. Bazzazan et al. also demonstrated that both free curcumin (Cur) and UIO-66-encapsulated curcumin (UIO-66-Cur) effectively inhibited cancer cell migration in MDA-MB-231 and SKBR3 cells, with UIO-66-Cur exhibiting greater efficacy than Cur alone (Bazzazan et al. 2023). The results obtained from this study showed the therapeutic use of UiO-66 nanocarrier containing letrozole in breast cancer cells. According to these studies, clinical studies on animal and human models are necessary to confirm the effect of nanoparticles. The conducted study showed an acceptable therapeutic effect of UiO-66 nanocarrier containing letrozole in laboratory conditions of breast cancer models. Therefore, the use of UiO-66 nanocarrier containing letrozole can be effective in increasing the expression of some pro-apoptotic genes. Based on this research and previous research, it can be concluded that the UiO-66 nanocarrier containing letrozole has strong anti-cancer effects on cancer cells, and the derivatives of this compound can play an essential role in treating this type of cancer in the future. Therefore, if the clinical process of this nanoparticle is confirmed, these nanoparticles can be used in clinical cases for breast cancer patients in the future.

In a study by Azandaryani et al., letrozole-incorporated folate-conjugated polymer-lipid hybrid nanoparticles showed the efficiency of the coupled folic acid carrier for the intracellular uptake of letrozole on the breast cancer line. Results showed that the entrapment and therapeutic efficiency of letrozole in the amphiphilic carrier were increased using the lipid nanoparticles and the surface modification, respectively (Hemati Azandaryani et al. 2017). In a study carried out by Hasanain. et al., the results demonstrated improved antitumor activity of Epirubicin (Epr) when encapsulated in pH-sensitive and folic acid-functionalized apoferritin drug carriers (HsAFr-Epr-FA complex). Results represent the use of FA to functionalize HsAFr could enhance the cellular uptake efficiency via FA-receptor-mediated endocytosis (Gomhor et al. 2018). In another study carried out by Abbas. et al., Apoferritin (AFr) functionalized with folic acid (FA) was used to encapsulate DOX to create the targeted protein nano complexes (TPNs). The second drug, MTO, was loaded into the cationic solid lipid nanoparticles (cSLN) to form the liposomal drug nanocomplex particles (MTO-cSLNs). Two complexes were then assembled by tight coupling through ionic interactions to obtain the final drug delivery system, the dual-targeted protein-lipid nano complexes (DTPLNs). Results confirmed that the DTPLNs display desired time-dependent and pH-dependent drug release behaviors. Results also demonstrated the improved anti-cancer efficacy of DOX and MTO in their encapsulated DTPLNs as compared to their free forms (Amer Ridha et al. 2021).

By simulating the complexity of the natural extracellular matrix, our research can provide a new opportunity to print vascular tissues and organs. However, the immunogenic aspect of using Nio-Tyro@CS-AL should be further considered to better understand treatment approaches for biological properties, angiogenesis, and wound healing. Also, more studies are needed to investigate the efficiency of a combination of these two procedures for skin tissue engineering. Additionally, due to the poor mechanical properties of the inks, the stability of these biological constructs after printing is not sufficient. Moreover, optimizing polymer concentrations for achieving suitable rheological properties of bioink is challenging. Future studies can be conducted by using niosomes loaded with different drugs with a positive effect on wound healing could be promising. Also, considering the great positive potentials of platelet concentrates and bioactive glasses in wound healing using combinational organic-inorganic nanocomposites consisting of drug-loaded niosomes, plasma rich in growth factor and bioactive glass could be a promising strategy in wound healing.

### Electronic supplementary material

Below is the link to the electronic supplementary material.

Supplementary Material 1

## Data Availability

Raw data supporting the findings of this study are available upon reasonable request from the corresponding author.
