# Supplementary material for: Preparation of UiO-66 loaded Letrozole nano-drug delivery system: enhanced anticancer and apoptosis activity
Source: AMB Express. 2024 Apr 15;14:38. doi: 10.1186/s13568-024-01689-1 (PMC11018590; doi:10.1186/s13568-024-01689-1)
Supplement: Supplementary file 1 — Supplementary Material 1 [file 13568_2024_1689_MOESM1_ESM.docx]

**Table S1.** **FTIR results of Let, UIO-66, and UIO-66-Let**

| Letrozole | 800 | Out of N-H plane |
| --- | --- | --- |
|  | 1000-1350 | N - C |
|  | 1550 - 1640 | N - H |
|  | 1600 - 1680 | C-C |
|  | 1640 - 1690 | N = C |
|  | 2250 | C ≡ N |
|  | 1450 -1600 | C = C |
| UIO-66 | 1505 | in- and out-of-phase  stretching modes of the carboxylate group |
|  | 1583 | C–C in  the aromatic compound of the organic linker |
|  | 1401 | C–O root in C–OH of carboxylic  acid |
|  | 670 and 749 | O–H bending and Zr–O modes |
|  | 553 | Zr–(OC) symmetric stretching |
|  | 498 | Zr–(OC) asymmetric stretching |
| UIO-66- Letrozole | 2232 | C ≡ N |
